# Swab‐based anal cancer screening in men living with HIV: Projected outcomes for different screening algorithms

**DOI:** 10.1002/ijc.70046

**Published:** 2025-08-05

**Authors:** Kirsten Rozemeijer, Fernando Dias Gonçalves Lima, Esther J. Kuyvenhoven, Henry J. C. de Vries, Renske D. M. Steenbergen, Jan M. Prins, Matthijs L. Siegenbeek van Heukelom

**Affiliations:** ^1^ Department of Pathology Amsterdam UMC, Vrije Universiteit Amsterdam Amsterdam The Netherlands; ^2^ Cancer Center Amsterdam Imaging and Biomarkers Amsterdam The Netherlands; ^3^ Department of Dermatology Amsterdam UMC, University of Amsterdam Amsterdam The Netherlands; ^4^ Department of Internal Medicine Amsterdam UMC, University of Amsterdam Amsterdam The Netherlands; ^5^ Center for Sexual Health, Department of Infectious Diseases Public Health Service Amsterdam Amsterdam The Netherlands; ^6^ Amsterdam Institute for Global Health and Development Amsterdam The Netherlands; ^7^ Amsterdam Institute for Infection and Immunity Infectious Diseases Amsterdam The Netherlands

**Keywords:** anal cancer, cancer screening, cytology, human papillomavirus viruses, test taking strategies

## Abstract

Screening for and treatment of anal cancer precursor lesions, high‐grade squamous intraepithelial lesions (HSIL), can prevent anal cancer. Recent guidelines set by the International Anal Neoplasia Society recommend digital anal rectal examination (DARE) and anal swab‐based screening of high‐risk individuals by means of high‐risk (hr)HPV testing or cytology. We used our biobank containing data of more than 600 high‐resolution anoscopy (HRA) screened participants (94% men with HIV) to compare the possible screening algorithms. We selected the 298 screening participants in whom anal swabs were successfully tested for hrHPV and cytology, parallel to HRA screening (DARE followed by complete visual inspection by HRA). We compared outcomes of several strategies (single‐test, co‐testing, two‐step testing) with one or two positive tests required for HRA referral, resulting in 20 possible screening algorithms. We also assessed the sensitivity of DARE to detect anal cancer. We found that the percentage of missed HSIL was lowest with hrHPV testing, either alone (14.2%) or combined with cytology (≥ASCUS threshold: 4.4%; HSIL threshold: 8.8%) (co‐testing or two‐step testing, with ≥1 positive test required for HRA referral). Using these screening algorithms, 61.0 %, 79.0 %, and 63.7% of the participants were referred for HRA. While in some scenarios a small percentage of cancers was missed, all were detected by DARE. Whatever strategy is chosen, screening outcomes will have to be monitored closely to evaluate the program and make adaptations when necessary.

## What's New?

Screening, combined with treatment of precancerous lesions, is key to preventing anal cancer. Here, the authors compare different screening algorithms. The International Anal Neoplasia Society recommends swab‐based screening as a first step, either cytology‐based or HPV‐based, and digital anal rectal exam (DARE). The authors assembled a biobank containing more than 600 paired swabs and biopsies, with which they compared the performance of different screening algorithms. High‐risk HPV testing, either alone or combined with cytology, missed the fewest lesions. These results can help guide the implementation of anal cancer screening programs that best suit the available resources.

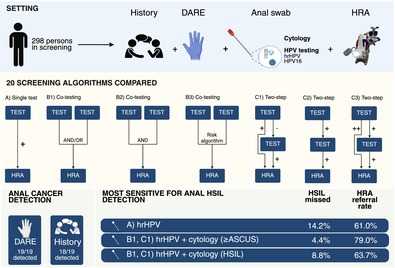

**Abbreviations**
AIN: anal intraepithelial neoplasia
ASCUS: atypical squamous cells of undetermined significance
DARE: digital anal rectal examination
FFPE: formalin‐fixed and paraffin‐embedded
HPV: human papillomavirus
HRA: high‐resolution anoscopy
hrHPV: high‐risk human papillomavirus
HSIL: high‐grade squamous intraepithelial lesion
IANS: International Anal Neoplasia Society
LAST: Lower Anogenital Squamous Terminology
LSIL: low‐grade squamous intraepithelial lesion
MSMLWH: men who have sex with men living with HIV
PLWH: people living with HIV
TWLWH: transgender women living with HIV

## 1 INTRODUCTION

Anal cancer incidence is rising, with men who have sex with men and living with HIV (MSMLWH) having the highest risk.^1^, ^2^ Human papillomavirus (HPV) infection, predominantly HPV type 16, causes anal cancer, which is preceded by a high‐grade squamous intraepithelial lesion (HSIL), also known as anal intraepithelial neoplasia (AIN) 2 or 3. Screening for and treatment of anal HSIL can lower the risk of anal cancer.^3^ In addition, with screening, anal cancers are detected in an earlier stage, resulting in better survival.^4^

The gold standard to detect precursor lesions is high‐resolution anoscopy (HRA), with biopsies taken of suspicious lesions. However, HRA‐based screening is costly, difficult to perform, and burdensome for participants. Furthermore, HRA capacity in many places is limited. Therefore, the International Anal Neoplasia Society (IANS) recently published guidelines on anal cancer screening recommending swab‐based screening as a first step.^5^ Proposed screening tests, prior to HRA, are anal cytology, high‐risk HPV (hrHPV) testing (with or without genotyping), and the digital anal rectal exam (DARE). Possible strategies included single‐testing, co‐testing (simultaneously 2 tests are performed), and two‐step testing (consecutively 2 tests are performed). The guidelines do not favor one particular strategy, and available data on test performance across other risk groups is limited, while there is a lack of data on longitudinal performance.

During the past 7 years, we have assembled a biobank containing paired anal swabs and biopsies of more than 600 screened participants. These participants were regularly screened (i.e., DARE, followed by anal cytology and HRA with intra‐anal biopsies taken from anal abnormalities) and in parallel, an anal swab was taken (i.e., before the complete visual inspection by HRA). hrHPV and cytology testing were performed on a subset of these swabs. This allowed us to compare in retrospect the performance of 20 possible screening algorithms (step‐by‐step procedures used to assess participants) of anal swab‐based screening followed by HRA in case of a positive test, as recommended by the IANS guidelines. For this total of 20 algorithms, we calculated and compared, for our screening population per 100 screening participants: the number of missed histologically proven anal HSIL and anal cancers; the number of cytology and hrHPV tests performed; and HRA referral rates. Finally, we assessed the sensitivity and specificity of history taking and DARE for HRA‐detected anal cancers.

## 2 MATERIALS AND METHODS

### 2.1 Study participants and biobank

At the HRA clinic of the department of Dermatology, Amsterdam UMC, MSMLWH and transgender women living with HIV (TWLWH) over 35 years, and persons referred because of anal symptoms are screened for anal HSIL. Screening consists of DARE followed by complete visual inspection by HRA, with intra‐anal biopsies taken from suspicious lesions according to previous IANS guidelines.^6^ Starting January 2018, all screening participants were asked to participate in our biobank study. Age, sex, gender, HIV status, and HRA screening history are collected from the electronic patient records. During their regular screening visit, an anal swab is taken before the complete visual inspection by HRA. Cytology and hrHPV testing were performed on a subset of these anal swabs as part of a cross‐sectional study on the accuracy of swabs.^7^ To date, clinical data, swabs, and biopsies of more than 600 screening participants are included in the biobank, including HRA‐detected anal cancers. Fourteen cancers were diagnosed at baseline (i.e., moment of inclusion in the biobank), of which four were recurrent cancers, and five additional participants had a history of anal cancer prior to inclusion in the biobank. For participants with recurrent anal cancer and participants with a history of anal cancer, data was also abstracted from the prior medical record.

### 2.2 Testing strategies and screening algorithms

The possible strategies consisted of single‐testing (one test is performed), co‐testing (simultaneously two tests are performed), and two‐step testing (consecutively two tests are performed). With co‐testing and two‐step testing, the number of positive tests required for a HRA referral can vary (at least one positive test required, two positive tests required, or risk stratification), resulting in a total of 7 strategies. In each strategy, various tests can be used (hrHPV, HPV16, and cytology with different thresholds), resulting in 20 possible screening algorithms. See Table 1 and Supplementary Figure 1 for a schematic overview of the different strategies and algorithms. No machine learning or artificial intelligence algorithms were used.

**TABLE 1:** Description of seven anal swab‐based testing strategies and 20 possible screening algorithms.

Test strategies	Screening algorithms	Indication for HRA referral
A. Single test	hrHPV	Positive result
	HPV16	
	Cytology (threshold ≥ASCUS)	
	Cytology (threshold HSIL)	
B1. Co‐testing	hrHPV + cytology (threshold ≥ASCUS)	≥ 1 positive result
	hrHPV + cytology (threshold HSIL)	
B2. Co‐testing	hrHPV + cytology (threshold ≥ASCUS)	2 positive results
	hrHPV + cytology (threshold HSIL)	
B3. Co‐testing	hrHPV (with genotyping) + cytology	HPV16 OR HSIL OR hrHPV positive (non16) + ≥ASCUS
C1. Two‐step testing	Step 1: hrHPV, step 2: cytology (threshold ≥ASCUS)	HRA referral if one or both tests are positive; second test if the first test is negative
	Step 1: hrHPV, step 2: cytology (threshold HSIL)	
	Step 1: Cytology (threshold ≥ASCUS), step 2: hrHPV	
	Step 1: Cytology (threshold HSIL), step 2: hrHPV	
C2. Two‐step testing	Step 1: hrHPV, step 2: cytology (threshold ≥ASCUS)	HRA referral if both tests are positive; second test if the first test is positive
	Step 1: hrHPV, step 2: cytology (threshold HSIL)	
	Step 1: Cytology (threshold ≥ASCUS), step 2: hrHPV	
	Step 1: Cytology (threshold HSIL), step 2: hrHPV	
C3. Two‐step testing	Step 1: hrHPV (genotyping), step 2: cytology (threshold ≥ASCUS)	HPV16 OR hrHPV positive (non16) + ≥ASCUS
	Step 1: hrHPV (genotyping), step 2: cytology (threshold HSIL)	HPV16 OR hrHPV positive (non16) + HSIL
	Step 1: Cytology, step 2: hrHPV	HSIL OR ≥ ASCUS + hrHPV positive

Abbreviations: ASCUS, atypical squamous cells of undetermined significance; HRA, high‐resolution anoscopy; hrHPV, high‐risk human papillomavirus; HSIL, high‐grade squamous intraepithelial lesions.

### 2.3 Procedures and assays

#### 2.3.1 Collection of the material

The anal swab specimen was collected by inserting a moistened Dacron swab and slowly retracting it while rotating and applying firm lateral pressure. Thereafter, the swab was transferred to a vial with 20 mL of ThinPrep medium (Hologic, Bedford, MA, USA) and swirled vigorously to release material.

#### 2.3.2 Cytology testing

Liquid‐based cytology was performed as part of regular care and graded according to the Bethesda System criteria and reporting terms.^8^, ^9^

#### 2.3.3 DNA isolation

4 mL of the (residual) collected ThinPrep solution was used for DNA isolation. DNA was isolated using the NucleoMag 96 DNA isolation kit (Macherey‐Nagel, Düren, Germany) and a Microlab Star robotic system (Hamilton, Gräfelfing, Germany) according to the manufacturer's recommendations.

#### 2.3.4 hrHPV testing

Swabs were tested for hrHPV as described previously using the GP5+/6 +—PCR EIA followed by Luminex typing or the QIAscreen HPV PCR Test (QIAgen, Venlo The Netherlands; an equivalent of the HPV‐Risk assay).^10^, ^11^, ^12^ Both tests are clinically validated for cervical screening purposes according to the international guidelines for HPV test requirements and perform equally.^13^, ^14^

#### 2.3.5 Histopathology

Anal biopsies were formalin‐fixed and paraffin‐embedded (FFPE) and examined by experienced pathologists. Low‐grade squamous intraepithelial lesions (LSIL; AIN1) and HSIL (AIN2‐3) were diagnosed and reported in accordance with criteria, terminology, and recommendations of the Lower Anogenital Squamous Terminology (LAST) project, where p16immunohistochemistry was used to support the AIN diagnosis if indicated.

### 2.4 Data analysis

#### 2.4.1 Main analysis

**FIGURE 1:**
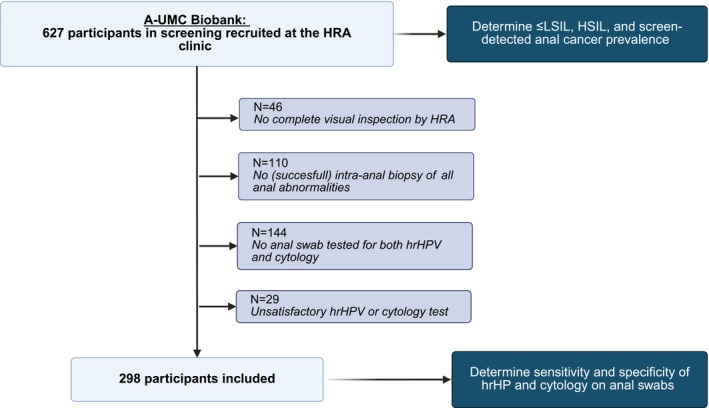
Flowchart from our screening population. Created with BioRender.com. HRA, high‐resolution anoscopy; hrHPV, high‐risk human papillomavirus; HSIL, high‐grade squamous intraepithelial lesions; LSIL, low‐grade squamous intraepithelial lesion.

First, we assessed the prevalence of no dysplasia or LSIL (≤LSIL), HSIL, and HRA‐detected anal cancer at baseline (i.e., moment of inclusion in the biobank). The biopsy with the highest histological grade was used as the study endpoint. Second, we determined the detection of histologically proven ≤LSIL, HSIL, and anal cancer with testing on anal swabs with hrHPV, HPV16, cytology (≥ASCUS), and cytology (HSIL). For the current analysis, we only included screening participants in whom the swab was successfully tested on both cytology and hrHPV. Furthermore, we excluded participants if there was no complete intra‐anal visualization by HRA or if no biopsies were taken of all suspicious lesions visualized by HRA, to avoid misclassification of the histological diagnosis (Figure 1). Third, we combined these data, and we were able to calculate per screening algorithm per 100 screening participants: the number of missed anal histologically proven HSIL and HRA‐detected anal cancers; the number of cytology and hrHPV tests required; the number of screening participants referred for HRA.

In sensitivity analyses, analyses were restricted to participants who were treated for HSIL in the past, to participants in whom the simultaneously obtained cytology results were available before histology was performed, and finally assuming a 50% lower anal cancer prevalence.

### 2.5 Sensitivity and specificity of history taking and DARE for detection of HRA‐detected anal cancers

To assess the sensitivity of history taking and DARE to detect anal cancer, we identified all participants with HRA‐detected anal cancer who took part in our biobank study. Their electronic patient records were screened for anal symptoms and DARE findings. We calculated the sensitivity of history taking and physical examination. In case of a recurrence, the first diagnosed HRA‐detected cancer was selected. To assess the specificity, we included all participants of whom the presence of physical symptoms and DARE findings were recorded in the biobank. We excluded participants if there was no complete intra‐anal visualization by HRA or if no biopsies were taken of all suspicious lesions visualized by HRA to avoid misclassification of the histological diagnosis.

### 2.6 Software

Statistical analyses and calculations were performed with Excel version 2408.

## 3 RESULTS

### 3.1 Study population

Per August 10, 2024, 627 screening participants were included in our Amsterdam UMC biobank. 95.7% were males, 3.3% were females, and 1.0% were transwomen (Table 2). 95.4% of participants were people living with HIV (PLWH) and it was estimated that 89.2% were MSMLWH. Of the participants, 29.2% were screened for the first time, and 49.1% were previously treated for HSIL. At the moment of inclusion, 2.2% (14 participants) had anal cancer, 29.0% had HSIL, 11.5% LSIL, and 57.3% had no dysplasia detected by HRA.

**TABLE 2:** Characteristics of biobank participants at the moment of inclusion, Amsterdam UMC 2018–2024.

	Population of the Amsterdam UMC biobank	Population of the Amsterdam UMC biobank with an hrHPV and cytology test on anal swabs	Population of the Amsterdam UMC biobank with history taking and DARE
	Used to determine prevalence of ≤LSIL, HSIL, and HRA‐detected anal cancer	Used to assess performance of hrHPV testing and cytology	Used to assess performance of history taking and DARE
*n*	627	298	439
Participant characteristics			
Age, median (range)	53 (26–83)	53 (29–83)	53 (29–83)
Gender			
Male	600 (95.7)	294 (98.7)	417 (95.0)
Female	21 (3.3)	3 (1.0)	17 (3.9)
Transwomen	6 (1.0)	1 (0.3)	5 (1.1)
HIV status			
Positive	598 (95.4)	291 (97.7)	417 (95.0)
Negative	27 (4.3)	7 (2.3)	21 (4.8)
Unknown	2 (0.3)	0 (0.0)	1 (0.2)
First‐time screeners	179 (29.2)^a^	72 (25.0)^b^	139 (33.1)^c^
Treated in the past for HSIL	301 (49.1)^a^	150 (52.1)^b^	194 (46.2)^c^
Histopathological diagnosis			
No dysplasia	359 (57.3)	139 (46.6)	226 (51.5)
LSIL	72 (11.5)	36 (12.1)	50 (11.4)
HSIL	182 (29.0)	113 (37.9)	144 (32.8)
HRA‐detected cancer^d^	14 (2.2)	10 (3.4)	19 (4.3)

*Note*: Numbers are *n* (%) unless stated otherwise.

Abbreviations: DARE, digital anal rectal examination; HRA, high‐resolution anoscopy; hrHPV, high‐risk human papillomavirus; HSIL, high‐grade squamous intraepithelial lesions; LSIL, low‐grade squamous intraepithelial lesions.

^a^ Screening history was unknown for 14 screening participants.

^b^ Screening history was unknown for 10 screening participants.

^c^ Screening history was unknown for 19 screening participants.

^d^ For 14 participants, anal cancer was detected at the moment of inclusion in the biobank. Five additional participants had a history of anal cancer prior to inclusion in the biobank.

To avoid any misclassification, 156 out of 627 participants were excluded due to incomplete intra‐anal visualization (*n* = 46) or because no biopsies were taken of all suspicious lesions visualized by HRA (*n* = 110). To assess the test performances of hrHPV (with and without genotyping) and cytology testing, an extra 173 out of the remaining 471 screening participants were excluded because swab results were incomplete (i.e., not tested for both hrHPV and cytology) (*n* = 144) or invalid results were obtained (*n* = 29), leaving 298 participants (Figure 1). To assess the performances of history taking and DARE, an extra 32 out of the remaining 471 screening participants were excluded because complete results of history taking or DARE results could not be retrieved from the electronic patient records, leaving 439 participants (Table 2).

### 3.2 Performance of the swab‐based screening algorithms

#### 3.2.1 Main analysis

The screening algorithms showing the lowest percentage of HSIL missed were:

- hrHPV testing (strategy A);
- hrHPV with cytology (≥ASCUS or HSIL threshold) co‐testing or two‐step testing (strategies B1 + C1: at least one of both tests need to be positive);
- Cytology (≥ASCUS and HSIL threshold) with hrHPV two‐step testing (strategy C1: at least one of both tests need to be positive).

With these algorithms only 4.4%–14.2% of HSIL were missed, corresponding with 1.3–4.1 HSIL per 100 screening participants, assuming a 29% HSIL prevalence. In 61.0%–79.0% of the participants, HRA referral would be indicated (Table 3).

**TABLE 3:** Number of anal high‐grade squamous intraepithelial lesions (HSIL) and anal cancers missed, number of tests taken, and high‐resolution anoscopy (HRA) referral rates for 7 strategies and 20 screening algorithms: Projected outcomes per 100 screening participants.^a^

	Numbers of lesions missed per 100 screening participants		Number of tests per 100 screening participants		
	*n* (%) of HRA‐detected HSIL missed^a^ (prevalence 29%)	*n* (%) of HRA‐detected anal cancer missed^a^ (prevalence 2%)	Cytology tests	hrHPV tests (with or without genotyping)	HRA referral rate
Strategy A: Single testing HRA referral if test is positive					
HPV16	18.5 (63.7%)	0.6 (30.0%)	0	100	19.0
hrHPV	4.1 (14.2%)	0.6 (30.0%)	0	100	61.0
Cytology (≥ASCUS)	5.4 (18.6%)	0.2 (10.0%)	100	0	61.7
Cytology (HSIL)	15.7 (54.0%)	0.6 (30.0%)	100	0	20.3
Strategy B1: Co‐testing (≥1) HRA referral if one or both tests are positive					
hrHPV + cytology (≥ASCUS)	1.3 (4.4%)	0.0 (0%)	100	100	79.0
hrHPV + cytology (HSIL)	2.6 (8.8%)	0.2 (10.0%)	100	100	63.7
Strategy B2: Co‐testing (2) HRA referral if both tests are positive					
hrHPV + cytology (≥ASCUS)	8.2 (28.3%)	0.8 (40.0%)	100	100	43.7
hrHPV + cytology (HSIL)	17.2 (59.3%)	1.0 (50.0%)	100	100	17.5
Strategy B3: Co‐testing (risk stratification) HRA referral if one of the screening tests indicates high risk or if both tests indicates intermediate risk					
hrHPV (genotyping) + cytology^b^	7.7 (26.5%)	0.2 (10.0%)	100	100	47.2
Strategy C1: Two‐step testing (≥1) HRA referral if one or both tests are positive; second test if the first test is negative					
hrHPV + cytology (≥ASCUS)	1.3 (4.4%)	0.0 (0%)	39.0	100	79.0
hrHPV + cytology (HSIL)	2.6 (8.8%)	0.2 (10.0%)	39.0	100	63.7
Cytology (≥ASCUS) + hrHPV	1.3 (4.4%)	0.0 (0%)	100	38.3	79.0
Cytology (HSIL) + hrHPV	2.6 (8.8%)	0.2 (10.0%)	100	79.7	63.7
Strategy C2: Two‐step testing (2) HRA referral if both tests are positive; second test if the first test is positive					
hrHPV + cytology (≥ASCUS)	8.2 (28.3%)	0.8 (40.0%)	61.0	100	43.7
hrHPV + cytology (HSIL)	17.2 (59.3%)	1.0 (50.0%)	61.0	100	17.5
Cytology (≥ASCUS) + hrHPV	8.2 (28.3%)	0.8 (40.0%)	100	61.7	43.7
Cytology (HSIL) + hrHPV	17.2 (59.3%)	1.0 (50.0%)	100	20.3	17.5
Strategy C3: Two‐step testing (risk stratification) HRA referral if the first test indicates high risk or if the first test indicates intermediate risk and the second test is positive; second test if the first test indicates intermediate risk					
hrHPV (genotyping) + cytology (≥ASCUS)^c^	7.4 (25.7%)	0.6 (30.0%)	42.0	100	46.2
hrHPV (genotyping) + cytology (HSIL)^d^	12.1 (41.6%)	0.6 (30.0%)	42.0	100	29.0
Cytology + hrHPV^e^	8.5 (29.2%)	0.4 (20.0%)	100	41.4	44.6

Abbreviations: ASC‐H, atypical squamous cells of undetermined significance cannot exclude HSIL; ASCUS, atypical squamous cells of undetermined significance; HRA, high‐resolution anoscopy; hrHPV, high‐risk human papillomavirus; HSIL, high‐grade squamous intraepithelial lesions; LSIL, low‐grade squamous intraepithelial lesions.

^a^ Prevalence was set equal to the prevalence as found in the Amsterdam UMC biobank: ≤LSIL prevalence 69%, HSIL prevalence 29%, HRA‐detected anal cancer prevalence 2%. Test performances were based on 175 ≤ LSIL, 113 HSIL, and 10 anal cancers.

^b^ HRA referral if hrHPV test is positive for HPV16 (regardless of cytology outcome), if cytology outcome is HSIL or ASC‐H (regardless of hrHPV outcome), or if hrHPV test is positive (non16) combined with an ASCUS or LSIL cytology outcome.

^c^ Immediate HRA referral if hrHPV test is positive for HPV16; also HRA referral if hrHPV test is positive (non16) combined with ASCUS or worse cytology outcome; second test if hrHPV test is positive (non16).

^d^ Immediate HRA referral if hrHPV test is positive for HPV16; also HRA referral if hrHPV test is positive (non16) combined with HSIL cytology; second test if hrHPV test is positive (non16).

^e^ Immediate HRA referral if cytology outcome is HSIL or ASC‐H; also HRA referral if cytology outcome is ASCUS or LSIL combined with a positive hrHPV test; second test if cytology outcome is ASCUS or LSIL.

The screening algorithms without missing any anal cancers were:

- hrHPV with cytology (≥ASCUS) co‐testing or two‐step testing (strategies B1 + C1: at least one of both tests need to be positive);
- Cytology (≥ASCUS) with hrHPV two‐step testing (strategy C1: at least one of both tests need to be positive).

With these algorithms, in 79.0% of the participants, HRA referral would be indicated.

The screening algorithms with high specificity, and thus low HRA referral rates, were (Supplementary Table 1):

- HPV16 testing (strategy A);
- Cytology (HSIL threshold) (strategy A);
- hrHPV with cytology (HSIL threshold) co‐testing or two‐step testing (strategies B2 + C2: both tests need to be positive);
- hrHPV (genotyping) with cytology (HSIL threshold) two‐step risk stratification testing (C3: hrHPV test need to be positive for HPV16 or positive [non16] combined with HSIL cytology).

HRA referral rate in these algorithms ranged between 17.5% and 29.0%. This was at the expense of missing 30 to 50% of the cancers and missing 41.6%–63.7% of the HSIL (Table 3).

When focusing on the total number of hrHPV (with or without genotyping) and cytology tests needed, all algorithms of strategy A required per 100 screening participants 100 tests. All algorithms of strategy B required 200 tests, and algorithms of strategy C required between 120.3 and 179.7 tests.

### 3.3 Sensitivity analyses

When restricting analyses to participants with a previous HSIL treatment, a higher percentage of HSIL were missed for all algorithms (+0.1 to +10.3 percentage points) except for single hrHPV screening (−1.7 percentage points), while the prevalence of HSIL decreased by 5 percentage points (i.e., 24 instead of 29%). As a result, the change in numbers of HSIL missed ranged between −2.4 and + 1.6 per 100 screening participants. For instance, with single cytology (≥ASCUS threshold) testing, 28.6% of HSIL were missed as compared to 18.6% before, corresponding with an increase of +10.0 percentage points. Combined with the 5 percentage points decrease in HSIL prevalence, this led to missing 7.0 HSIL per 100 participants with a previous HSIL treatment, compared to 5.4 per 100 in the overall group of participants, corresponding to an increase of 1.6 HSIL missed per 100 participants (i.e., +1.6). Because ≤LSIL detection also changed by −2.6 to +2.9, and ≤LSIL prevalence increased by 4 percentage points, the change in HRA referral rates ranged between −5.6 and +0.9 per 100 screening participants. Algorithms with the largest change in HRA referral rates were single cytology testing (strategy A1) (≥ASCUS threshold, −5.6 percentage points; HSIL threshold; −4.5 percentage points) and hrHPV with cytology co‐testing or two‐step testing (strategies B2 + C2: both tests need to be positive) (≥ASCUS threshold, −3.2 percentage points; HSIL threshold; −3.9 percentage points). The relative performance of the strategies and screening algorithms was unaffected (Supplementary Table 2).

When restricting analyses to HSIL cases where cytology was performed before histology results were available (*n* = 204), no relevant differences in detection rates were observed either. The change in numbers of HSIL missed ranged between −0.9 and + 2.6 per 100 screening participants while the change in HRA referral rates ranged between −3.8 and −1.2. The relative performance of the strategies and screening algorithms was unaffected (data not shown).

When we decreased the anal cancer prevalence in the screened population by 50%, effects were minimal. HRA referral rates decreased by at most 0.6 per 100 screening participants, and the relative performance of the strategies and screening algorithms remained similar (data not shown).

### 3.4 Sensitivity and specificity of history taking and DARE to detect anal cancer with HRA

Overall, in 19 participants in the Amsterdam UMC biobank, anal cancer was detected with HRA screening. For 14 participants, anal cancer was detected at the moment of inclusion (i.e., at baseline); five additional participants had a history of anal cancer prior to inclusion (Table 2). Of these 19 participants with HRA‐detected anal cancer, 18 reported symptoms (sensitivity 94.7%). Symptoms were anal pain, anal blood loss, a perceived lump in or around the anal canal, constipation or a changed stool pattern, mucus in stool, incontinence, itching, weight loss, and tenesmus (Table 4, Supplementary Table 3). All participants with anal cancer had palpable abnormalities at DARE consistent with anal cancer (100.0%); 13 reported pain during DARE, and 6 had blood loss during DARE (Table 4, Supplementary Table 4).

**TABLE 4:** Sensitivity and specificity of history taking and DARE, in 19 screening participants with anal cancer and 420 screening participants without anal cancer.

	History taking		DARE	
	Positive	Negative	Positive	Negative
HRA‐detected anal cancer	18/19 (94.7%)	1/19 (5.3%)	19/19 (100%)	0/19 (0.0%)
No HRA‐detected anal cancer	101/420 (24.0%)	319/420 (76.0%)	85/420 (20.2%)	335/420 (79.8%)

*Note*: No HRA‐detected anal cancer: no dysplasia (*n* = 226), LSIL (*n* = 50), or HSIL (*n* = 144).

Abbreviations: DARE, digital anal rectal examination; HRA, high‐resolution anoscopy.

The specificity of reported complaints during history taking was 76.0%. The specificity of abnormal findings during DARE was 79.8% (Table 4, Supplementary Tables 3 and 4). The combined specificity of DARE plus history taking was 64.5% (data not shown).

## 4 DISCUSSION

When focusing on test sensitivity to detect HSIL, all screening algorithms of strategies B1 (co‐testing) and C1 (two‐step testing) were promising: hrHPV combined with cytology (≥ASCUS or HSIL threshold). Only 4.4%–8.8% of HSIL were missed, while the percentage of screening participants referred to HRA ranged between 63.7% and 79.0%. The total number of hrHPV and cytology tests needed for 100 screening participants were for strategy B1 (co‐testing) 200 tests, and for strategy C1 (two‐step testing) 138.3 to 179.7 tests. With hrHPV only testing (strategy A), more HSIL were missed (14.2%), but only 61.0% of screening participants would be referred for HRA.

When focusing on test sensitivity to detect anal cancer, hrHPV combined with cytology (≥ASCUS threshold) co‐testing or two‐step testing (strategies B1 + C1) was most promising. However, performing DARE also detected all cancers.

From the 19 participants with screen‐detected cancers in this study, all had abnormalities at DARE (sensitivity of 100%) and all except one presented with anal cancer symptoms. This underlines the importance of DARE and history taking for the detection of cancer. These findings are in line with Berry et al.^15^ At the same time, the specificity of history taking and DARE for anal cancer were 76% and 80%, while the combined specificity was 65%.

The HSIL detection rates of cytology (≥ASCUS or HSIL threshold), HPV16, and hrHPV testing are in line with those reported in two previous systematic reviews.^16^, ^17^ Moreover, our finding that the use of cytology triage after a positive hrHPV test can reduce HRA referral rates corresponds with recent Australian and American findings.^18^, ^19^

It is estimated that 10% of anal cancers are independent of hrHPV.^20^ Out of the ten cases of anal cancer with complete swab results (i.e., both hrHPV and cytology tested), three tested negative for hrHPV on their swabs. Careful examination revealed that hrHPV was actually present in one of these swabs, but below the detection threshold. In the second person with anal cancer, no hrHPV was detected in either the swab or biopsy, indicating that this was an hrHPV‐independent cancer. In the third person with anal cancer, hrHPV was detected in the biopsy but not in the swab, suggesting that hrHPV was undetectable or absent in the swab material collected.

The IANS guidelines indicate several swab‐based screening strategies: hrHPV‐based, cytology‐based, or a combination of these. The data presented here can help identify the screening strategy that best suits the local situation. In many centers, including our own, capacity to perform cytology is limited. In that case, the following options remain: HPV16 testing (strategy A); hrHPV testing, either alone (strategy A) or combined with cytology two‐step testing (ASCUS or HSIL threshold) (strategy C1); hrHPV (genotyping) with cytology (ASCUS or HSIL threshold) two‐step risk stratification testing (strategy C3). When HRA capacity is also limited, HPV16 testing (strategy A) or hrHPV (genotyping) with cytology (HSIL threshold) two‐step risk stratification testing (strategy C3) is the best option.

The strength of this study is that we are the first to combine all strategies and algorithms of the recently published guidelines^5^ with real‐time data of screening participants in whom hrHPV and cytology testing on anal swabs had been performed in combination with the “gold standard” HRA screening. This enabled us to calculate the effect on HSIL detection, anal cancer detection, and HRA referral rate for the various screening algorithms proposed by the guidelines.

A limitation of this study is that it concerns a Dutch screening population which is restricted to MSMLWH and TWLWH. Nonetheless, the HSIL prevalence of 29% in MSMLWH is comparable with that of international studies.^21^ The prevalence of HRA‐detected cancers was relatively high (i.e., 2.2%), which could be explained by referrals of participants with anal symptoms. In addition, it is possible that participants with anal cancer were more likely to sign informed consent to be included in our biobank. However, we showed in a sensitivity analysis that this high cancer prevalence affected the results only minimally. When we decreased the anal cancer prevalence in the screened population by 50%, HRA referral rates decreased at most by 0.6 per 100 screening participants, and the relative performance of the strategies and screening algorithms remained similar. Next, our screening population consisted of 25.0% first‐time screeners. Due to small sample sizes, we were unable to perform a sensitivity analysis restricted to this group. Although we expect the relative performance of the screening algorithms in first‐time screeners to remain unchanged, an expectation supported by analyses restricted to participants with a history of HSIL treatment, it is possible that the performance of certain screening tests and algorithms may be influenced by differences in the screening population. Therefore, more longitudinal research is warranted. Third, it is possible that cytology outcome was not always assessed independently of histology. However, different staff members in our department report histology and cytology outcomes, and when restricting analyses to cases where cytology was performed before histology results were available, we found similar outcomes. Fourth, to avoid misclassification, we excluded all participants where there was the slightest doubt that a lesion was missed. For instance, we excluded a participant with an AIN1 diagnosis in whom five intra‐anal biopsies were taken, but approximately 20% of the anal canal could not be inspected. Finally, this study is based on calculations of cross‐sectional data. This fits the purpose of our study, that is, estimating the short‐term effect in order to make an informed decision about which screening test strategy to choose. More longitudinal research is needed to assess the long‐term harms and benefits and implications of each screening algorithm. Additional research could be conducted using alternative techniques, such as methylation testing.

In this data‐driven strategy paper, we calculated and compared outcomes of the various possible screening strategies and algorithms of anal swab‐based screening as proposed by the 2024 guidelines of the International Anal Neoplasia Society, using data of a real‐world screening population. The data presented here can help identify the screening strategy that best suits the local situation. Nevertheless, performing a DARE and/or history taking should be the cornerstone of every screening program. Whatever strategy is chosen, screening outcomes will have to be monitored closely to evaluate the program and make adaptations when necessary.

## AUTHOR CONTRIBUTIONS

**Kirsten Rozemeijer:** Formal analysis; investigation; writing – original draft; conceptualization; data curation; writing – review and editing; visualization. **Fernando Dias Gonçalves Lima:** Data curation; writing – original draft; writing – review and editing; visualization; investigation. **Esther J. Kuyvenhoven:** Data curation; writing – review and editing. **Henry J. C. de Vries:** Conceptualization; writing – review and editing. **Renske D. M. Steenbergen:** Conceptualization; writing – review and editing. **Jan M. Prins:** Investigation; conceptualization; writing – original draft; writing – review and editing; supervision. **Matthijs L. Siegenbeek van Heukelom:** Investigation; writing – original draft; writing – review and editing; supervision; conceptualization.

## FUNDING INFORMATION

KR was supported by a grant from Stichting Life Sciences & Health ‐ TKI, and FDGL was supported by the Amsterdam Academic Medical Centres PhD Scholarship. The source of funding did not have any influence on the design of the study, collection, analysis, interpretation of the data, writing of the manuscript, or the decision to submit the article for publication.

## CONFLICT OF INTEREST STATEMENT

KR received consultancy fees from FUJIREBIO paid to her institution. RDMS is a minority stockholder of Self‐screen B.V., a spin‐off company of VUmc, which owns patents on methylation markers and HPV detection. RDMS received consultancy fees from AstraZeneca paid to her institution. HJCdV received financial compensation or goods for research from Medigene, Gilead, and MSD; financial compensation for presentations from Abbott and Janssen; and financial compensation for advice to Medigene and Novartis. All other authors report no potential conflicts.

## ETHICS STATEMENT

We adhered to the Declaration of Helsinki and the Code of Conduct for Responsible Use of Left‐over Material of the Dutch Federation of Biomedical Scientific Societies. All participants gave written informed consent. Ethical approval was waived by the Institutional Review Board of the Amsterdam UMC, location AMC, under reference number 18/316 (paired anal swabs and biopsies of AIN) and under number 18/333 (swabs of anal cancer).

## DISCLOSURE

The contents of this manuscript have not been copyrighted or published previously. However, test performances of hrHPV and cytology, which were used as input for this simulation study, were derived from swabs of which a subset was used in our recently published paper.^7^ This paper focused on the performance of methylation analysis to identify patients with advanced HSIL with an increased cancer risk.

## Supporting information


**Supplementary table 1.** Sensitivity, specificity, false positives, positive predictive value, and negative predictive value of 7 strategies and 20 screening algorithms.^a^
**Supplementary table 2**. Sensitivity analyses: Restricting detection to 150 screening participants with a previous HSIL treatment: Number of anal HSIL missed, number of tests.
**Supplementary table 3**. Sensitivity and specificity of history taking.
**Supplementary table 4**. Sensitivity and specificity of DARE.
**Supplementary Figure 1**. Seven testing strategies and 20 screening algorithms described in more detail.

## Data Availability

All source code is publicly available on GitHub (https://github.com/KRozemeijer/Projected-Outcomes-for-Different-Screening-Algorithms.git). Further information is available from the corresponding author upon request.
