# Supplementary material for: Swab‐based anal cancer screening in men living with HIV: Projected outcomes for different screening algorithms
Source: Int J Cancer. 2025 Aug 5;157(11):2259–68. doi: 10.1002/ijc.70046 (PMC12496001; doi:10.1002/ijc.70046)
Supplement: Supplementary file 1 — Supplementary table 1. Sensitivity, specificity, false positives, positive predictive value, and negative predictive value of 7 strategies and 20 screening algorithms.a Supplementary table 2. Sensitivity analyses: Restricting detection to 150 screening participants with a previous HSIL treatment: Number of anal HSIL missed, number of tests. Supplementary table 3. Sensitivity and specificity of history taking. Supplementary table 4. Sensitivity and specificity of DARE. Supplementary Figure 1. Seven testing strategies and 20 screening algorithms described in more detail. [file IJC-157-2259-s001.pdf]

Swab-Based Anal Cancer Screening in men living with HIV: Projected Outcomes for Different Screening Algorithms

Kirsten Rozemeijer, Fernando Dias Gonçalves Lima, Esther J. Kuyvenhoven, Henry J.C. de Vries, Renske D.M. Steenbergen, Jan M. Prins, Matthijs L. Siegenbeek van Heukelom

Table of content

| Table of content | Page |
|---|---|
| Supplementary Table 1 | 2-4 |
| Supplementary Table 2 | 5-7 |
| Supplementary Table 3 | 8 |
| Supplementary Table 4 | 9 |
| Supplementary Figure 1 | 10 |

Supplementary table 1. Sensitivity, specificity, false positives, positive predictive value, and negative predictive value of 7 strategies and 20 screening algorithms[a]

| | Sensitivity (% of ≥HSIL, detected) | Specificity (% of ≤LSIL, not detected) | False positives (% of ≤LSIL, detected) | Positive predictive value (% of HRA referrals, ≥HSIL) | Negative predictive value (% of not HRA referrals, ≤LSIL) |
|---|---|---|---|---|---|
| Strategy A: Single testing<br>*HRA referral if test is positive* | | | | | |
| HPV16 | 39.0 | 89.7 | 10.3 | 62.7 | 76.4 |
| hrHPV | 84.6 | 49.7 | 50.3 | 43.1 | 87.9 |
| Cytology (≥ASCUS) | 82.1 | 47.4 | 52.6 | 41.2 | 85.4 |
| Cytology (HSIL) | 48.0 | 92.0 | 8.0 | 72.8 | 79.6 |
| Strategy B1: Co-testing (≥1)<br>*HRA referral if one or both tests are positive* | | | | | |
| hrHPV + cytology (≥ASCUS) | 95.9 | 28.6 | 71.4 | 37.6 | 93.9 |
| hrHPV + cytology (HSIL) | 91.1 | 48.6 | 51.4 | 44.3 | 92.4 |
| Strategy B2: Co-testing (2)<br>*HRA referral if both tests are positive* | | | | | |
| hrHPV + cytology (≥ASCUS) | 70.7 | 68.6 | 31.4 | 50.3 | 84.0 |
| hrHPV + cytology (HSIL) | 41.5 | 93.1 | 6.9 | 73.0 | 77.9 |
| Strategy B3: Co-testing (risk stratification)<br>*HRA referral if one of the screening tests indicates high risk or if both tests indicates intermediate risk* | | | | | |
| [b]hrHPV (genotyping) + cytology | 74.8 | 65.1 | 34.9 | 49.0 | 85.1 |
| Strategy C1: Two-step testing (≥1)<br>*HRA referral if one or both tests are positive; second test if the first test is negative* | | | | | |
| hrHPV + cytology (≥ASCUS) | 95.9 | 28.6 | 71.4 | 37.6 | 93.9 |

| | | | | | |
|---|---|---|---|---|---|
| hrHPV + cytology (HSIL) | 91.1 | 48.6 | 51.4 | 44.3 | 92.4 |
| Cytology (≥ASCUS) + hrHPV | 95.9 | 28.6 | 71.4 | 37.6 | 93.9 |
| Cytology (HSIL) + hrHPV | 91.1 | 48.6 | 51.4 | 44.3 | 92.4 |
| Strategy C2: Two-step testing (2)<br>*HRA referral if both tests are positive; second test if the first test is positive* | | | | | |
| hrHPV + cytology (≥ASCUS) | 70.7 | 68.6 | 31.4 | 50.3 | 84.0 |
| hrHPV + cytology (HSIL) | 41.5 | 93.1 | 6.9 | 73.0 | 77.9 |
| Cytology (≥ASCUS) + hrHPV | 70.7 | 68.6 | 31.4 | 50.3 | 84.0 |
| Cytology (HSIL) + hrHPV | 41.5 | 93.1 | 6.9 | 73.0 | 77.9 |
| Strategy C3: Two-step testing (risk stratification)<br>*HRA referral if the first test indicates high risk or if the first test indicates intermediate risk and the second test is positive; second test if the first test indicates intermediate risk* | | | | | |
| [c]hrHPV (genotyping) + cytology (≥ASCUS) | 74.0 | 66.3 | 33.7 | 49.7 | 85.0 |
| [d]hrHPV (genotyping) + cytology (HSIL) | 59.3 | 84.6 | 15.4 | 63.3 | 82.2 |
| [e]Cytology + hrHPV | 71.5 | 67.4 | 32.6 | 49.6 | 84.0 |

[a]Prevalence was set equal to the prevalence as found in the Amsterdam UMC biobank: ≤LSIL prevalence 69%, HSIL prevalence 29%, HRA-detected anal cancer prevalence 2%. Test performances were based on 175 ≤LSIL, 113 HSIL, and 10 anal cancers.

[b]HRA referral if hrHPV test is positive for HPV16 (regardless of cytology outcome), if cytology outcome is HSIL or ASC-H (regardless of hrHPV outcome), or if hrHPV test is positive (non16) combined with an ASCUS or LSIL cytology outcome.

[c]Immediate HRA referral if hrHPV test is positive for HPV16; also HRA referral if hrHPV test is positive (non16) combined with ASCUS or worse cytology outcome; second test if hrHPV test is positive (non16).

[d]Immediate HRA referral if hrHPV test is positive for HPV16; also HRA referral if hrHPV test is positive (non16) combined with HSIL cytology; second test if hrHPV test is positive (non16).

[e]Immediate HRA referral if cytology outcome is HSIL or ASC-H; also HRA referral if cytology outcome is ASCUS or LSIL combined with a positive hrHPV test; second test if cytology outcome is ASCUS or LSIL.

Abbreviations: ASCUS: atypical squamous cells of undetermined significance; HRA: high-resolution anoscopy; hrHPV: high-risk human papillomavirus; HSIL: high-grade squamous intraepithelial lesions; LSIL: low-grade squamous intraepithelial lesions.

Supplementary table 2. Sensitivity analyses: Restricting detection to 150 screening participants with a previous HSIL treatment: Number of anal HSIL missed, number of tests taken, and high-resolution anosopy (HRA) referral rates for 7 strategies and 20 screening algorithms: Projected outcomes per 100 screening participants[a]

| | Numbers of lesions missed per 100 screening participants | Number of tests per 100 screening participants | | |
|---|---|---|---|---|
| | n (%) of HRA-detected HSIL missed [a](prevalence 24%) | Cytology tests | hrHPV tests (with or without genotyping) | HRA referral rate |
| Strategy A: Single testing<br>*HRA referral if test is positive* | | | | |
| HPV16 | 16.1 (66.1%) | 0 | 100 | 19.2 |
| hrHPV | 3.1 (12.5%) | 0 | 100 | 61.9 |
| Cytology (≥ASCUS) | 7.0 (28.6%) | 100 | 0 | 56.1 |
| Cytology (HSIL) | 15.7 (64.3%) | 100 | 0 | 15.7 |
| Strategy B1: Co-testing (≥1)<br>*HRA referral if one or both tests are positive* | | | | |
| hrHPV + cytology (≥ASCUS) | 1.3 (5.4%) | 100 | 100 | 77.6 |
| hrHPV + cytology (HSIL) | 2.2 (8.9%) | 100 | 100 | 64.0 |
| Strategy B2: Co-testing (2)<br>*HRA referral if both tests are positive* | | | | |
| hrHPV + cytology (≥ASCUS) | 8.7 (35.7%) | 100 | 100 | 40.4 |
| hrHPV + cytology (HSIL) | 16.6 (67.9%) | 100 | 100 | 13.6 |
| Strategy B3: Co-testing (risk stratification)<br>*HRA referral if one of the screening tests indicates high risk or if both tests indicates intermediate risk* | | | | |
| [b]hrHPV (genotyping) + cytology | 7.0 (28.6%) | 100 | 100 | 46.0 |
| Strategy C1: Two-step testing (≥1) | | | | |

| | | | | |
|---|---|---|---|---|
| *HRA referral if one or both tests are positive; second test if the first test is negative* | | | | |
| hrHPV + cytology (≥ASCUS) | 1.3 (5.4%) | 38.1 | 100 | 77.6 |
| hrHPV + cytology (HSIL) | 2.2 (8.9%) | 38.1 | 100 | 64.0 |
| Cytology (≥ASCUS) + hrHPV | 1.3 (5.4%) | 100 | 43.9 | 77.6 |
| Cytology (HSIL) + hrHPV | 2.2(8.9%) | 100 | 84.3 | 64.0 |
| Strategy C2: Two-step testing (2)<br>*HRA referral if both tests are positive; second test if the first test is positive* | | | | |
| hrHPV + cytology (≥ASCUS) | 8.7 (35.7%) | 61.9 | 100 | 40.4 |
| hrHPV + cytology (HSIL) | 16.6 (67.9%) | 61.9 | 100 | 13.6 |
| Cytology (≥ASCUS) + hrHPV | 8.7 (35.7%) | 100 | 56.1 | 40.4 |
| Cytology (HSIL) + hrHPV | 16.6 (67.9%) | 100 | 15.7 | 13.6 |
| Strategy C3: Two-step testing (risk stratification)<br>*HRA referral if the first test indicates high risk or if the first test indicates intermediate risk and the second test is positive; second test if the first test indicates intermediate risk* | | | | |
| [c]hrHPV (genotyping) + cytology (≥ASCUS) | 7.8 (32.1%) | 42.7 | 100 | 43.9 |
| [d]hrHPV (genotyping) + cytology (HSIL) | 11.3 (46.4%) | 42.7 | 100 | 27.1 |
| [e]Cytology + hrHPV | 7.8 (32.1%) | 100 | 40.4 | 42.5 |

[a]Prevalence was set equal to the prevalence as found in the Amsterdam UMC biobank for this population (i.e., screening participants with a previous HSIL treatment): ≤LSIL prevalence 73% and HSIL prevalence 24%. Since previous HSIL treatment data were not recorded for HRA-detected anal cancers, their prevalence could not be determined. Therefore, HRA-detected anal cancer prevalence was assumed to be equal to that of the total screening population (i.e., 2%). Test performances were based on 94 ≤LSIL and 56 HSIL. Test performance on HRA-detected anal cancer were set equal to that of the screening population (i.e., Table 3).

[b]HRA referral if hrHPV test is positive for HPV16 (regardless of cytology outcome), if cytology outcome is HSIL or ASC-H (regardless of hrHPV outcome), or if hrHPV test is non HPV16 positive combined with an ASCUS or LSIL cytology outcome.

[c]Immediate HRA referral if hrHPV test is positive for HPV16; also HRA referral if hrHPV test is positive (non16) combined with ASCUS or worse cytology outcome; second test if hrHPV test is positive (non16).

[d]Immediate HRA referral if hrHPV test is positive for HPV16; also HRA referral if hrHPV test is positive (non16) combined with HSIL cytology; second test if hrHPV test is positive (non16).

[e]Immediate HRA referral if cytology outcome is HSIL or ASC-H; also HRA referral if cytology outcome is ASCUS or LSIL combined with a positive hrHPV test; second test if cytology outcome is ASCUS or LSIL.

Abbreviations: ASC-H: atypical squamous cells of undetermined significance cannot exclude HSIL; ASCUS: atypical squamous cells of undetermined significance; HRA: high-resolution anoscopy; hrHPV: high-risk human papillomavirus; HSIL: high-grade squamous intraepithelial lesions.

Supplementary table 3. Sensitivity and specificity of history taking

| | Presenting symptoms | | | | |
|---|---|---|---|---|---|
| | Anal pain | Anal blood loss | Constipation | Other | Any |
| No dysplasia (n=226) | 12 (5.3%) | 16 (7.1%) | 7 (3.1%) | 37 (16.4%) | 47 (20.8%) |
| LSIL (n=50) | 2 (4.0%) | 4 (8.0%) | 2 (4.0%) | 14 (28.0%) | 15 (30.0%) |
| HSIL (n=144) | 5 (3.5%) | 13 (9.0%) | 6 (4.2%) | 32 (22.2%) | 39 (27.1%) |
| Total: No HRA-detected cancer (n=420) | 19 (4.5%) | 33 (7.9%) | 15 (3.6%) | 83 (19.8%) | 101 (24.0%) |
| HRA-detected anal cancer (n=19) | 14 (73.7%) | 13 (68.4%) | 7 (36.8%) | 15 (78.9%) | 18 (94.7%) |

Abbreviations: HRA: high-resolution anoscopy; HSIL: high-grade squamous intraepithelial lesions; LSIL: low-grade squamous intraepithelial lesions.

Supplementary table 4. Sensitivity and specificity of DARE

| | Abnormalities at DARE | | | |
|---|---|---|---|---|
| | Pain | Palpable abnormalities | Blood loss | Any |
| No dysplasia (n=226) | 4 (1.8%) | 25 (11.1%) | 16 (7.1%) | 41 (18.1%) |
| LSIL (n=50) | 1 (2.0%) | 10 (20.0%) | 3 (6.0%) | 13 (26.0%) |
| HSIL (n=144) | 1 (0.7%) | 20 (13.9%) | 11 (7.6%) | 31 (21.5%) |
| Total: No HRA-detected cancer (n=420) | 6 (1.4%) | 55 (13.1%) | 30 (7.1%) | 85 (20.2%) |
| HRA-detected anal cancer (n=19) | [a]13 (81.3%) | 19 (100.0%) | [b]6 (40.0%) | 19 (100.0%) |

[a]13 Out of 16. 3 Were unknown.

[b]6 Out of 15. 4 Were unknown.

Abbreviations: DARE: digital anal rectal examination; HRA: high-resolution anoscopy; HSIL: high-grade squamous intraepithelial lesions; LSIL: low-grade squamous intraepithelial lesions.

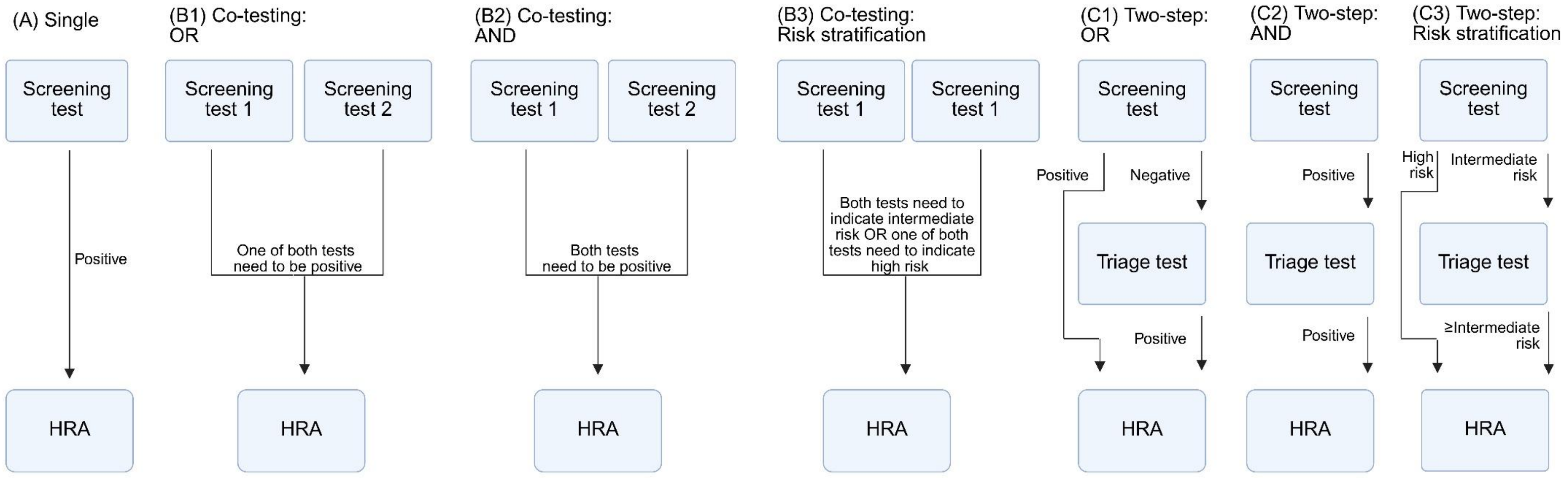


**Supplementary Figure 1. Seven testing strategies and 20 screening algorithms described in more detail.**

Screening strategies consist of single-testing (A), co-testing (OR situation (B1), AND situation (B2), and risk stratification (B3)), and two-step testing (OR situation (C1), AND situation (C2), and risk stratification (C3)). Screening tests consist of hrHPV (with or without genotyping) and cytology (both ≥ASCUS and HSIL threshold). Triage tests consist of hrHPV and cytology (both ≥ASCUS and HSIL threshold).

Created with BioRender.com

Abbreviations: HRA: high-resolution anoscopy.
